# Cancer

**Published:** 1906-03-03

**Authors:** 


### CANCER.

**Cancer of the Uterus.**—The early recognition of cancer of the uterus is a subject of the utmost importance. In the great majority of cases the victims of this common and fell disease present themselves for treatment too late for successful operative interference. And even although the profession are becoming increasingly alert in the matter of detecting it, a certain and early diagnosis is often attended by much difficulty. Sir Halliday Croom [1] in a recent paper lays stress on the value of curetting in certain cases as a diagnostic measure, and especially on the routine microscopic examination of the fragments removed in every case of curetting, no matter for what purpose this has been done. He gives some striking instances in which the microscope showed malignancy which could not have been suspected clinically. A very remarkable case is quoted which also emphasises the great value of hæmorrhage as an early symptom and the duty of medical practitioners in giving serious attention even to slight cases of uterine hæmorrhage which cannot be satisfactorily accounted for. The patient was a widow aged forty-seven, a nurse of large experience, whose only complaint was that two days previously a little hæmorrhage had followed the use of a vaginal douche. A most careful examination discovered nothing more than the slightest possible loss of epithelium on the anterior cervical lip. The patient, however, was not satisfied, and insisted on being curetted, and, to the operator's great surprise, the microscopist found that the specimen showed squamous epithelioma. Hysterectomy was speedily performed and further evidences of cancer found in the cervical canal. The significance of hæmorrhages at or near the menopause is not to be underrated, though that event is so frequently manifested by profuse bleeding, and hæmorrhage occurring after the cessation of menstruation demands in every case the careful exclusion of malignant disease, though it may be due to gouty conditions or to fibroids. Croom also emphasises the frequency with which malignant disease begins or progresses after labour, and upholds the practice of American authorities who advocate periodic examinations after confinement in order to detect the appearance of any lesions which may follow. In an instance quoted, a patient who had had an incomplete abortion two months previously, followed by persistent bleeding was curetted and the scrapings examined as a mere matter of routine. Malignant growth was reported and proved to be correct by the discovery in the fundus of the uterus after hysterectomy of a wellmarked carcinoma. On the other hand, confusion sometimes arises between cancer and the products of conception, and in not a few cases the patient's subsequent history has shown that the cancer declared by the microscopist to be present was not really so. Croom strongly advises more collaboration between the gynæcologist and the pathologist, and urges that notes of the clinical history should always accompany the specimen for the guidance of the latter. A fungating polypus has been mistaken microscopically and clinically for cancer and unnecessary hysterectomy performed. An excessively friable, readily bleeding, lobulated cervix is generally regarded, and rightly so, as typical of cervical cancer, yet this writer has known three such cases where the microscopic examination was negative and borne out by the subsequent well-being of the patients. The formation of a correct opinion is thus seen to be in many instances a matter of much difficulty. But in most cases a careful microscopic examination guided by a knowledge of the clinical history will suffice. A case of carcino sarcoma uteri has been described by Herbert Spencer.[2] The term is applied to the simultaneous presence of a carcinoma and a sarcoma in the uterus, while that of sarcoma carcinomatodes is reserved for malignant growths partly sarcomatous and partly carcinomatous in structure. In Spencer's case, he thought the sarcomatous tumour might have originated in a fibroid. Professor Wertheim[3] is an advocate for an extensive abdominal operation in many cases of uterine cancer regarded as inoperable by the vaginal route. In 179 such cases, where operation by the vagina would have been hopeless, 30 per cent. or more have been free from recurrence after five years. He reserves vaginal hysterectomy for early cases of cervical cancer and for growths in the body. The special features of Wertheim's abdominal operation are a very careful isolation of the wound area, extensive peritoneal reflections, careful isolation of the ureters, removal of the iliac glands and drainage by gauze packing through the vaginal wound. He has reduced the mortality of his operation recently from 15 to 7 per cent., but the mortality of such an extensive operation as he advocates would be very high in the hands of an operator who had not very large experience. The Professor has found recurrence more frequent in those cases in which he has not removed the glands along the iliac vessels.

[1] Edin. Med. Jour., August. [2] Lancet, Oct. 14, p. 1109. [3] Ibid., Aug. 5, p. 371.

